# Social network analysis in business and management research: A bibliometric analysis of the research trend and performance from 2001 to 2020

**DOI:** 10.1016/j.heliyon.2022.e09270

**Published:** 2022-04-12

**Authors:** Adhe Rizky Anugerah, Prafajar Suksessanno Muttaqin, Wahyu Trinarningsih

**Affiliations:** a Bioresource Management Lab, Institute of Tropical Forestry and Forest Products (INTROP), Universiti Putra Malaysia, 43400 Serdang, Selangor, Malaysia; b Department of Logistics Engineering, School of Industrial and System Engineering, Telkom University, 40257 Bandung, Indonesia; c Faculty of Economics and Business, Universitas Sebelas Maret, 57126 Surakarta, Indonesia

**Keywords:** Social network analysis, Bibliometrics, Clustering analysis, Business and management, Literature

## Abstract

In the past years, research in Social Network Analysis (SNA) has increased. Initially, the research area was limited to sociology and anthropology but has now been used in numerous disciplines. The business and management discipline has many potentials in employing the SNA approach due to enormous relational data, ranging from employees, stakeholders to organisations. The study aims to analyse the research trend, performance, and the utilisation of the SNA approach in business and management research. Bibliometric analysis was conducted by employing 2,158 research data from the Scopus database published from 2001 to 2020. Next, the research quantity and quality were calculated using Harzing's Publish or Perish while VOSviewer visualised research topics and cluster analysis. The study found an upward trend pattern in SNA research since 2005 and reached the peak in 2020. Generally, six subjects under the business and management discipline have used SNA as a methodology tool, including risk management, project management, supply chain management (SCM), tourism, technology and innovation management, and knowledge management. To the best of the authors' knowledge, the study is the first to examine the performance and analysis of SNA in the overall business and management disciplines. The findings provide insight to researchers, academicians, consultants, and other stakeholders on the practical use of SNA in business and management research.

## 1 Introduction

The SNA is a theory investigating the relations and interactions based on anthropology, sociology, and social psychology to assess social structures (Erçetin and Neyişci, 2014). The social structure in a network theory comprises individuals or organisations named nodes linked through one or more types of interdependencies, such as friendship, kinship, financial exchange, knowledge or prestige (Parell, 2012). The actors range across different levels, from individuals, web pages, families, large organisations, and nations. Nowadays, SNA usage has grown, utilised in anthropology and sociology and several fields of science, including business and management disciplines.

However, studies on the SNA trends and applications in business and management are limited. Although published articles provide a catalogue of SNA concepts, they lack explanatory mechanisms on its application (Borgatti and Li, 2009). Thus, the study aims to assess publication performances and explore SNA usage in business and management studies using Bibliometric analysis. The bibliometric methodology has been widely used to provide quantitative analysis of written publications using statistical tools (Ellegaard and Wallin, 2015). It can help detect established and emergent topical areas, research clusters and scholars, and others (Fahimnia et al., 2015). This analysis reveals important publications and objectively depicts the linkages between and among articles about a specific research topic or field by examining how frequently they have been co-cited by other published articles (Fetscherin and Usunier, 2012).

Bibliometric analysis has at least two primary objectives: 1) to quantitatively measure the quality of journals or authors using statistical indicators such as citations rates (Vieira et al., 2021), and 2) to analyse the knowledge structure and development of specific research fields (Jing et al., 2015). Hence, the study addresses the following research questions: RQ1: *What is the current research trend of SNA in business and management research*? RQ2: *What is the most productive year of SNA in the business and management discipline*? RQ3: *What are the most influential and productive institutions, authors, journals, and countries*? RQ4: *What is the use of SNA in the business and management discipline and their cluster topics in the past 20 years*?

Several literature reviews and bibliometric papers on the use of SNA in general business and management areas have been published, but the number is limited. Monaghan, Lavelle, & Gunnigle (2017) analysed SNA usage in management research and practice to discuss the critical dimensions for handling and analysing network data for business research. The authors discovered four dimensions in initial engagement with SNA in business and management research: structure of research design, data collection, handling of data and data interpretation. Nonetheless, studies did not explain the distribution of research clusters and how SNA can be used in practical business and management research. Specifically, Su et al. (2019) conducted a Bibliometric analysis on SNA literature with no limitation of subject discipline and collected the data from Web of Science (WoS), covering 20 publication years from 1999 to 2018. Nevertheless, Su et al. (2019) mainly discussed the SNA publication performance but not how the approach was used previously.

In the more specific subject area of business and management, SNA has been explored to unveil the relationship between organisations, as conducted by Sozen et al. (2009). The SNA has been used to measure the organisations' social capital, map resource dependency relations, and discover coalitions and cliques between organisations. Kurt and Kurt (2020) have explored the potential of SNA in international business (IB) research, because of two fundamental phemomena: firm internationalisation and multinational enterprises (MNEs). From the marketing perspective, SNA could detect the most influential actors to efficiently spread a message in online communities for marketing purposes (Litterio et al., 2017).

The current study conducted a clustering analysis to identify and analyse SNA performance and its application in general business and management discipline using bibliometrics information. Thus, academicians, managers, consultants, and other stakeholders could understand when and how to apply the SNA approach. For instance, SNA can identify potential risks contributing to schedule delays in project risk management (Li et al., 2016). The discussion section explores how SNA has been previously used in business and management research. Besides, the study addresses the problem in Borgatti and Li (2009), exploring the actual application of SNA in management and business research.

## 2 Methods

### 2.1 Data sources and search strategy

The primary study objective is to analyse the research trend and explore the SNA approach in business and management research. A Bibliometric analysis was employed due to its accuracy in quantifying and evaluating scientific publications (Carmona-Serrano et al., 2020). Additionally, the data were collected through the Scopus database. Although Scopus and WoS are the main and most comprehensive sources for Bibliometric analysis, Scopus has more advantages: more inclusive content coverage, more openness to society, and available individual profiles for all authors, institutions, and serial sources. Additionally, many papers have confirmed that Scopus provides wider overall coverage and Scopus indexing a greater amount of unique sources not covered by WoS (Pranckutė, 2021). In the business, economics, and management area, 89% of articles listed in WoS are listed in Scopus. Hence, the study area (business and management) chose the Scopus database for further analysis.

The next step involved determining the search string, including all documents with the title, abstract, and keywords containing "network analysis" or "Social Network Analysis". These two main keywords are representative enough to reach the objective; they are not too wide and specific. The main goal is the utilisation of SNA as a concept and as a methodology can be widely captured. These two versions of keywords “Social Network Analysis” and “Network Analysis” without the “social” has a significant impact. Some articles did not put the complete sentence of SNA, although the articles mainly discussed the concept of network analysis. One of the examples is the ownership structure related research developed by Vitali et al. (2011) which changed the word "social network analysis" to "corporate network analysis". The term “social” in SNA refers to people interaction, while in the operation research, the relationship could be between airport, stakeholders, corporation, etc.

In the first run, 113,945 research related to SNA was found in the Scopus database, mainly Engineering and Computer Science fields. Besides, the search results were limited to the subject area in business, management, and accounting and covered publication from 2001 to 2020 (20 years). The study also excluded non-journal articles, such as conference proceedings, trade reports, book chapters, and others.

**Figure 1:**
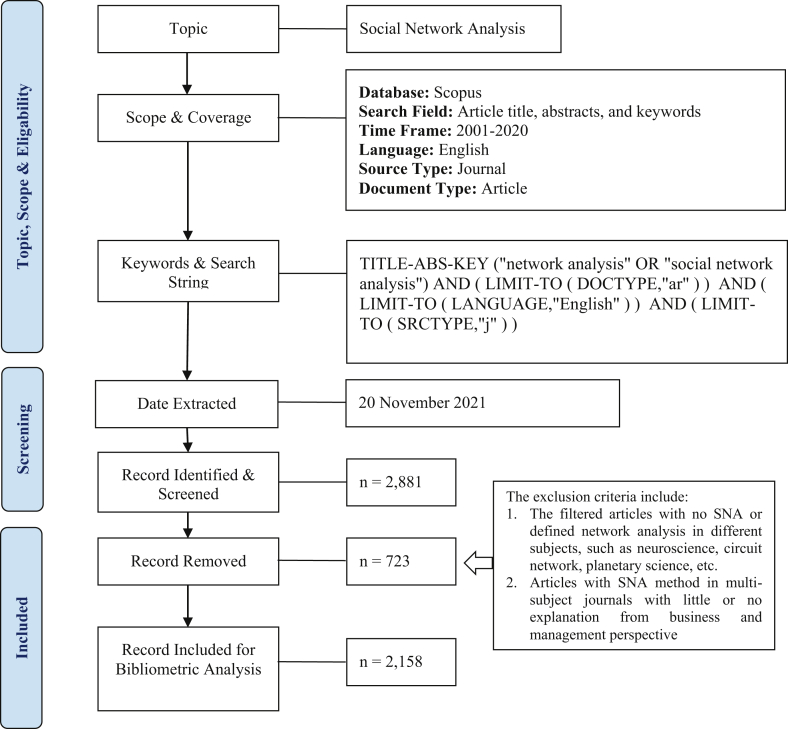
The search strategy flow diagram (adopted from (Zakaria et al., 2021)).

The search limitations have resulted in 2,881 articles, but many were still not related to network analysis or business and management. Further, 723 articles were excluded, covering articles in neuroscience, bibliometric, circuit network (engineering), earth and planetary science, chemistry, etc., although the articles employed network analysis as a methodology. The exclusion was also applied to articles that use SNA in multi-subject journals with little or no explanation in business, management, and accounting perspectives. One example is the Journal of Cleaner Production listed in four subject areas: business, management and accounting; energy; industrial and manufacturing engineering; and environmental science. In this journal, SNA theory is used to identify the relationship between ecosystems by measuring the flow of energy or material between organisms, which has little or no explanation from business and management perspectives. At the end of the search, 2,158 articles were extracted for further analysis. The flow chart on the data collection strategy is presented in Figure 1.

### 2.2 Data analysis

The first stage involved analysing the data descriptively to identify the quality and quantity using standard Bibliometric measures (Hirsch, 2005). The total number of publications (TP) assessed the quantity dimension, whereas other metrics assessed the quality dimension, such as total citation (TC), number of cited publications (NCP), average citation per publication (C/P), average citations per cited publication (C/CP) (Hirsch, 2007). Additionally, the g-index (*g*) and h-index (*h*) are usually included in the Bibliometric measures to predict future achievement rather than standard measures. The indicators are applied to various levels: country-level, organisation-level, journal-level, and author-level. The information is processed and analysed using Harzing publish or perish (PoP) software by extracting Research Information System (RIS) data from the Scopus database.

The second stage visualised the research network to understand the relationship between nodes, including authors, affiliations (organisations), countries, citations, and keywords. Nonetheless, only keywords co-occurrence was carried out to examine the past, current, and future potential of SNA in business and management research. The study analysed the keywords based on the frequency, edges, and clusters. The combination between nodes (keywords) and edges (the relationship between keywords) form clusters with numerous research themes (Dhamija and Bag, 2020). The bigger nodes show a higher occurrence in the keyword visualisations, and the thicker edges show the higher link strength. Meanwhile, cluster analysis in the study represents a set of similar keywords in one group, different in other groups to identify the research interest and keywords combination within the group. The cluster mapping was performed by VOSviewer, an open-access programme to construct and view Bibliometric maps (van Eck and Waltman, 2010). Besides, the study developed an overlay visualisation to explore research evolution in SNA over time.

## 3 Results

### 3.1 Description of retrieved literature

The study is limited to SNA research in business and management research published between 2001 to 2020. The study also excluded review papers, conference papers, editorials, and other documents besides journal articles for further analysis. Ultimately, the study retrieved a total of 2,158 articles. Although the search was limited to only English articles, the study identified seven bilingual articles in Spanish (3 articles), Chinese (2 articles), Lithuanian (1 article), and Portuguese (1 article). The articles had 58,522 citations, an average of 2,926 citations per year, and 27 citations per paper. The complete citation metrics for the articles are shown in Table 1.

**Table 1:** Citations metrics.

Metrics	Data
Papers	2,158
Number of Citations	58,522
Years	20
Average of Citations per Year	2,926.10
Average of Citations per Paper	27.12
Average of Authors per Paper	2.71
*h*-index	107
*g*-index	171

Besides SNA as a primary keyword, the top keywords were "innovation" (6.16%), "project management" (3.48%), "knowledge management" (3.29%), "decision making" (2.97%), "complex networks" (2.69%), and others. The top keywords are listed in Table 2. High-frequency keywords show the popularity of a specific topic (Pesta et al., 2018). The listed keywords in the study usually appear together in SNA and are used to explore the potential use of SNA in business and management research.

**Table 2:** Top keywords.

No	Keywords	Total Publication	Percentage (%)
1	Innovation	133	6.16%
2	Project Management	75	3.48%
3	Knowledge Management	71	3.29%
4	Decision Making	64	2.97%
5	Complex Networks	58	2.69%
6	Social Media	58	2.69%
7	China	55	2.55%
8	Tourist Destination	53	2.46%
9	Social Capital	51	2.36%
10	Data Mining	42	1.95%
11	Technological Development	41	1.90%
12	Construction Industry	40	1.85%
13	Patents and Inventions	40	1.85%
14	Stakeholder	39	1.81%
15	Air Transportation	37	1.71%
16	Numerical Model	37	1.71%
17	Communication	36	1.67%
18	Information Management	36	1.67%
19	Research	36	1.67%
20	Supply Chain Management	36	1.67%
21	Knowledge	35	1.62%
22	Internet	34	1.58%
23	Tourism	34	1.58%
24	Centrality	32	1.48%
25	Forecasting	32	1.48%

### 3.2 Research Growth

Although SNA research productivity presented the ups and downs throughout the year, a consistent upward trend was found in the pattern. Research in the first five years (2001–2005) was limited and never reached 30 publications per year. In 2002, only 10 articles were published, increasing almost five times in 2007 (n = 49 documents). The number increased until 2020, slightly decreasing in 2011, 2015, and 2019. Meanwhile, the most productive year was in 2020, with 334 published articles.

**Figure 2:**
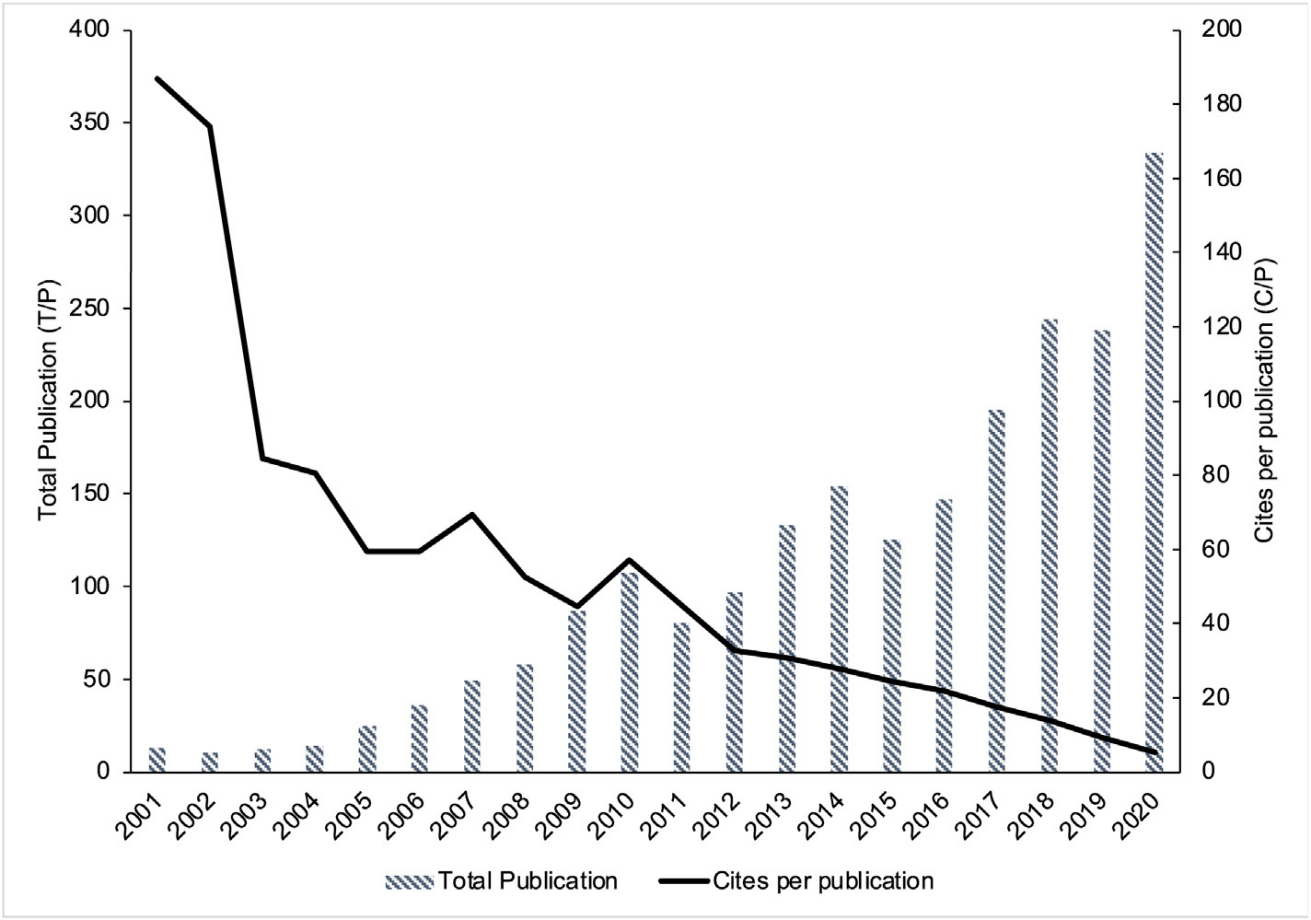
Total publications and citations by year.

The articles published in 2001 had the highest average citation per publication (c/p = 186.69). However, the highest *h-index* were in 2010 (*h =* 43) and 2014 (*h =* 36) which indicate high cumulative impact of the articles measured by its quantity with quality. Low citations per publication in recent years were expected due to increasing citation counts over time. The publication trend and average citations per publication are presented in Figure 2.

### 3.3 Top countries, institutions, and authors in SNA business and management research

The United States (US), the United Kingdom (UK), and China were the most prolific countries with 605, 230 and 215 articles, respectively. The study discovered no dominating continent that produced SNA business and management research, and all were equally distributed except for Africa. The top ten countries list (see Table 3) showed one North American, four Asian and Oceanian, and five European countries. As the top most productive countries, the United States and the United Kingdom published quality articles with highest average citation per publication of 39.34 and 32.80, respectively. Meanwhile, Asian countries had small citations (based on the top ten most productive countries): South Korea (c/p = 21.84) and China (c/p = 27.08) were at the bottom of the ranking according to the c/p calculation.

**Table 3:** The top ten countries contributed to the publications.

Country	TP	NCP	TC	C/P	C/CP	*h*	*g*
United States	605	580	23799	39.34	41.03	78	134
United Kingdom	230	217	7545	32.80	34.77	44	79
China	215	193	5226	24.31	27.08	41	64
Italy	181	174	4712	26.03	27.08	35	63
Australia	149	144	3958	26.56	27.49	33	57
South Korea	122	111	2424	19.87	21.84	25	44
Germany	105	100	2404	22.90	24.04	31	45
Spain	105	98	2978	28.36	30.39	26	51
Netherlands	75	72	2359	31.45	32.76	25	46
Taiwan	75	68	2034	27.12	29.91	24	43

Notes: TP = total number of publications; NCP = number of cited publications; TC = total citations; C/P = average citations per publication; C/CP = average citations per cited publication; h = h-index; and g = g-index.

Hong Kong Polytechnic University was the most productive institution with 45 published articles and had the highest *h-* and *g-*index (see Table 4). The publication number was higher than the second most productive, Università Bocconi (n = 25). However, the top ten list showed that the University of Arizona had the highest c/p with 102.47, followed by the University of Kentucky (c/p = 58.40) and the Università Bocconi (c/p = 58.36). As the most productive country, there were four United States universities listed as the top most productive institutions but it only covered 10.9% from the total US articles. This indicates that the publications were distributed to other US institutions. Nevertheless, articles from two Hong Kong insitutions, Hong Kong Polytechic University and City University Hongkong accumulated 57 articles (82.61% of the country's total articles).

**Table 4:** Top 10 most influential institutions in SNA (business and management) research.

Institution	Country	TP	NCP	TC	C/P	C/CP	*h*	*g*
Hong Kong Polytechnic University	Hong Kong	45	42	1173	26.07	27.93	18	33
Università Bocconi	Italy	25	25	1459	58.36	58.36	15	25
University of Central Florida	United States	19	19	594	31.26	31.26	10	19
The University of Queensland	Australia	19	19	852	44.84	44.84	12	19
University of Illinois Urbana-Champaign	United States	17	17	408	24.00	24.00	10	17
RMIT University	Australia	16	15	327	20.44	21.80	10	15
The University of Arizona	United States	15	14	1537	102.47	109.79	12	14
University of Kentucky	United States	15	15	876	58.40	58.40	12	15
Seoul National University	South Korea	14	13	350	25.00	26.92	9	13
Alliance Manchester Business School	United Kingdom	14	13	369	26.36	28.38	8	13

Notes: TP = total number of publications; NCP = number of cited publications; TC = total citations; C/P = average citations per publication; C/CP = average citations per cited publication; h = h-index; and g = g-index.

**Figure 3:**
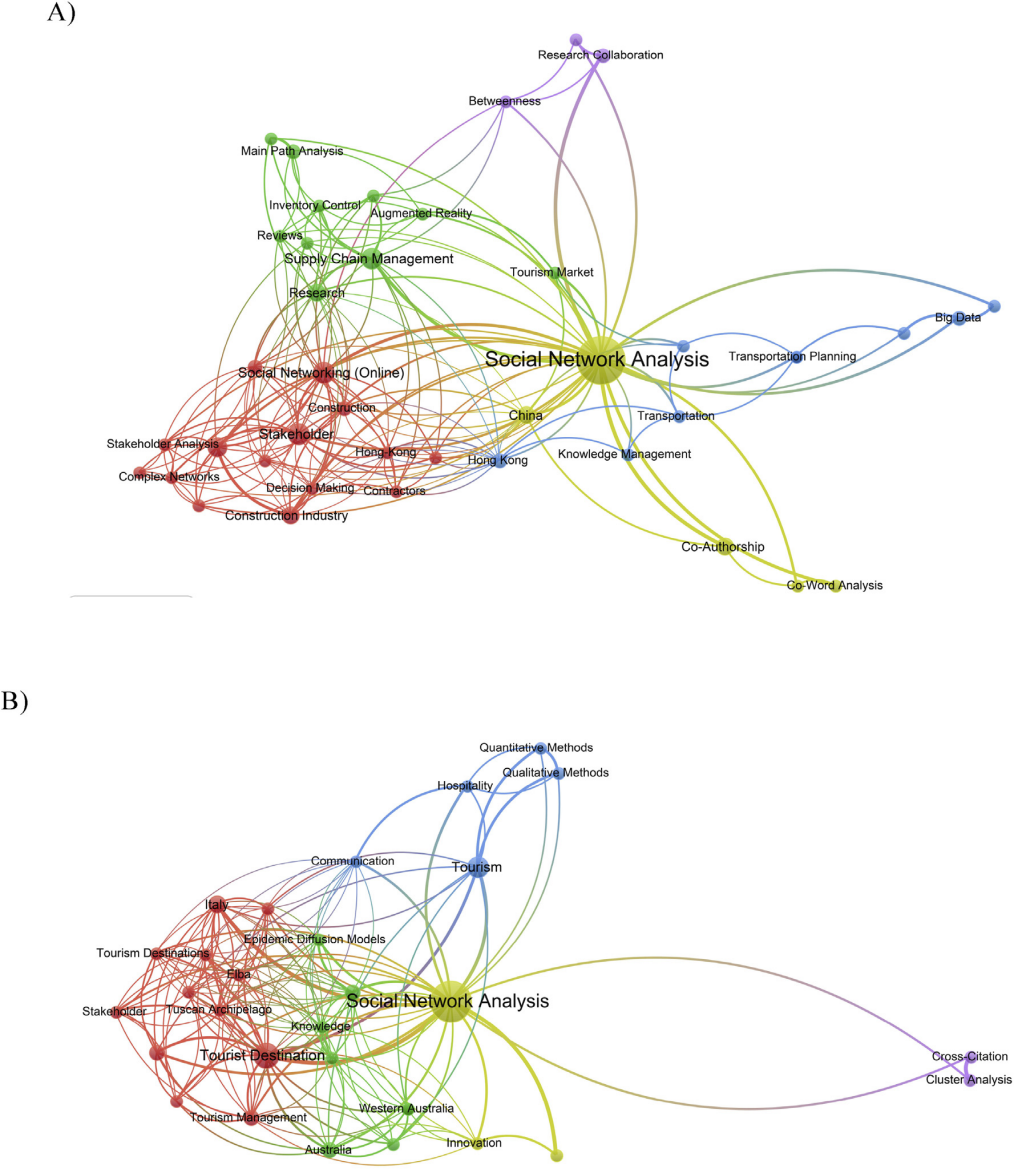
Research topic comparison between A) Hong Kong Polytechnic University and B) Università Bocconi.

The two most productive institutions conducted different research themes. Figure 3 shows that Hong Kong Polytechnic University use SNA in numerous areas, such as "supply chain management", "transportation", "big data", "knowledge management", “stakeholder analysis in construction project” and others. Nonetheless, Università Bocconi research mostly involved tourism; the keywords were "tourist destination", "tourism management", "stakeholder", and “hospitality”. Besides, 17 of the 25 articles (68.0%) from Università Bocconi were written by Rodolfo Baggio, the author with the most article in SNA business and management. Table 5 presents the most productive authors with at least six published articles in SNA.

**Table 5:** Most productive authors with a minimum of six articles.

Author Name	Affiliation	Country	TP	NCP	TC	C/P	C/CP	*h*	*g*
Baggio, R.	Università Bocconi	Italy	17	17	1127	66.29	66.29	12	17
Scott, N.	University of the Sunshine Coast	Australia	8	8	683	85.38	85.38	7	8
Fronzetti Colladon, A.	Università degli Studi di Perugia	Italy	7	7	124	17.71	17.71	5	7
Hossain, L.	University of Nebraska Kearney	United States	7	6	117	16.71	19.50	4	7
Kapucu, N.	University of Central Florida	United States	7	7	469	67.00	67.00	6	7
Casanueva, C.	Universidad de Sevilla	Spain	6	6	572	95.33	95.33	6	6
Grippa, F.	Northeastern University	United States	6	6	68	11.33	11.33	3	6
Shen, G.Q.	Hong Kong Polytechnic University	Hong Kong	6	6	257	42.83	42.83	4	6
Weng, C.S.	Takming University of Science and Technology	Taiwan	6	5	23	3.83	4.60	3	4
Yeo, G.T.	Incheon National University	South Korea	6	6	51	8.50	8.50	4	6

Notes: TP = total number of publications; NCP = number of cited publications; TC = total citations; C/P = average citations per publication; C/CP = average citations per cited publication; h = h-index; and g = g-index.

Rodolfo Baggio was the most productive author with the most publications, followed by Noel Scott (n = 8) from Australia and Andrea Fronzetti Colladon (n = 7) from Italy. Based on the average citations per document, Carlos Casanueva from the Universidad de Sevilla, Spain, had the highest score with an average of 95.33 citations per document. Baggio, R. and Scott, N, as the most productive authors have similar research interest, which is in tourism management. Based on the study database, they had written four articles together using a network analysis approach in tourism management. Fronzetti Colladon, A. is an expert in big data, creativity and innovation management while Hossain L., utilising SNA in organisational communication network during crisis or emergency events.

### 3.4 Most active journals

The majority of the articles were mostly published in Elsevier's journals. Specifically, seven out of 12 source titles were Elsevier's journals; two journals were published by the American Society of Civil Engineers (ASCE), two journals by Taylor's and Francis, and one by Emerald. The Journal of Technological Forecasting and Social Change was the most active source with 84 articles, followed by Transportation Research Part E: Logistics and Transportation Review and Knowledge based Systems with 45 and 44 articles, respectively. Based on the average citations per publication, Construction Management and Economics had the highest score (c/p = 78.50), followed by Decision Support Systems (c/p = 55.33) and Transportation Research Part E (c/p = 50.51). Table 6 demonstrates a list of the most active sources in publishing research in SNA (business and management) and its impact score (cite score and SCImago Journal Rank (SJR) 2019).

**Table 6:** Most active source title.

Source Title	Publisher	TP	C/P	Cite Score	SJR 2019
Transportation Research Part E Logistics and Transportation Review	Elsevier	45	50.51	9.3	2.04
Knowledge Based Systems	Elsevier	44	34.91	11.3	1.59
Decision Support Systems	Elsevier	40	55.33	10.5	1.56
Journal of Construction Engineering and Management	ASCE	34	34.94	6.4	0.97
Technology Analysis and Strategic Management	Taylor & Francis	33	15.03	4.1	0.76
Journal of Air Transport Management	Elsevier	25	14.44	6.5	1.22
Journal of Management In Engineering	ASCE	25	26.72	7.9	1.65
Journal of Business Research	Elsevier	24	19.33	9.2	2.05
Journal of Knowledge Management	Emerald	22	31.64	10.3	1.84
Construction Management and Economics	Taylor & Francis	20	78.50	5.6	0.88
International Journal of Project Management	Elsevier	20	45.95	16.4	2.76

Notes: TP = total number of publications; C/P = average citations per publication.

**Figure 4:**
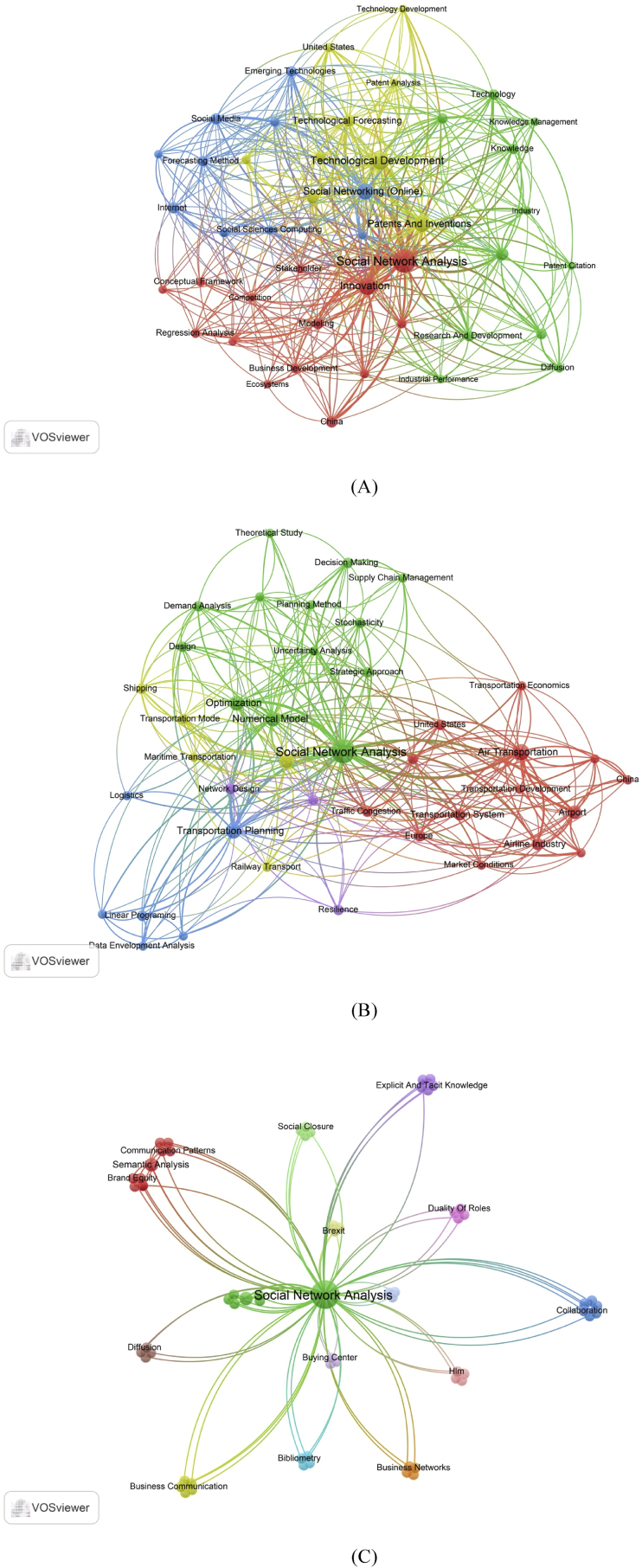
Most frequent keywords in a) Technological Forecasting and Social Change; b) Transportation Research Part E: Logistics and Transportation Review; and c) Journal of Business Research.

The SNA usage in business and management varies and depends on the journal scope. Particularly, SNA is used to study the interaction between people in the social environment and in various other subjects. The use of SNA in specific journals was explored by visualising the network of keywords relationship, as presented in Figure 4. The selected three journals publishing research in SNA (business and management) had a different perspective in employing SNA as a tool to analyse the relationship between nodes.

Journal of Technological Forecasting and Social Change primarily utilised "innovation", "technological development", "patents and inventions", "technology adoption", "emerging technology", and others. SNA can also be used for transportation research as published in Transportation Research Part E: Logistics and Transportation Review with keywords "numerical model", "transportation planning", "air transportation", "optimisation", "freight transport", and others. Meanwhile, research published in Journal of Business Research published articles with keywords “business networks”, “social closure”, “collaboration”, “diffusion”, and others. The SNA can also be used in construction and project management, tourism management, urban planning and development, and organisational study (coordination and competition).

### 3.5 Highly-cited articles

Tsai (2002) published the top-cited article in SNA business and management research titled "*Social structure of "coopetition" within a multiunit organisation: Coordination, Competition, and Intra organisational Knowledge Sharing"*. The publication had 1,124 or 56.2 citations per year. Besides, the article explored another use of SNA, explained in the "most active journals" section in the organisational study. The study revealed that most of the top 15 articles related to inter- or intra- organisational networks, and some papers explored the use of SNA in social communication, supply chain, tourism marketing, and others. Table 7 shows the top 15 highly-cited articles.

**Table 7:** Top 15 Highly-cited articles.

No	Authors	Title	Citation
1.	Tsai (2002)	The social structure of "coopetition" within a multiunit organisation: Coordination, competition, and intra organisational knowledge sharing	1,124
2.	Reagans and Zuckerman (2001)	Networks, Diversity, and Productivity: The Social Capital of Corporate R&D Teams	1009
3.	Brown et al. (2007)	Word of mouth communication within online communities: Conceptualising the online social network	926
4.	Provan and Milward (2001)	Do networks really work? A framework for evaluating public-sector organisational networks	829
5.	Stephen and Toubia (2010)	Deriving value from social commerce networks	501
6.	Pan et al. (2007)	Travel blogs and the implications for destination marketing	458
7.	Sosa et al. (2004)	The misalignment of product architecture and organisational structure in complex product development	439
8.	Boschma & ter Wal (2007)	Knowledge networks and innovative performance in an industrial district: The case of a footwear district in the South of Italy	414
9.	Hafner-Burton et al. (2009)	Network analysis for international relations	397
10.	Borgatti and Li (2009)	On Social Network Analysis in a supply chain context	390
11.	Kim et al. (2011)	Structural investigation of supply networks: A Social Network Analysis approach	389
12.	Nagurney et al. (2002)	A Supply Chain Network Equilibrium Model	364
13.	Netzer et al. (2012)	Mine Your Own Business: Market-structure Surveillance Through Text Mining	361
14.	Acedo et al. (2006)	Co-authorship in Management and Organizational Studies: an Empirical and Network Analysis	357
15.	Li and Wu (2010)	Using Text Mining and Sentiment Analysis for Online Forums Hotspot Detection and Forecast	342

### 3.6 The use of SNA in business and management research

**Figure 5:**
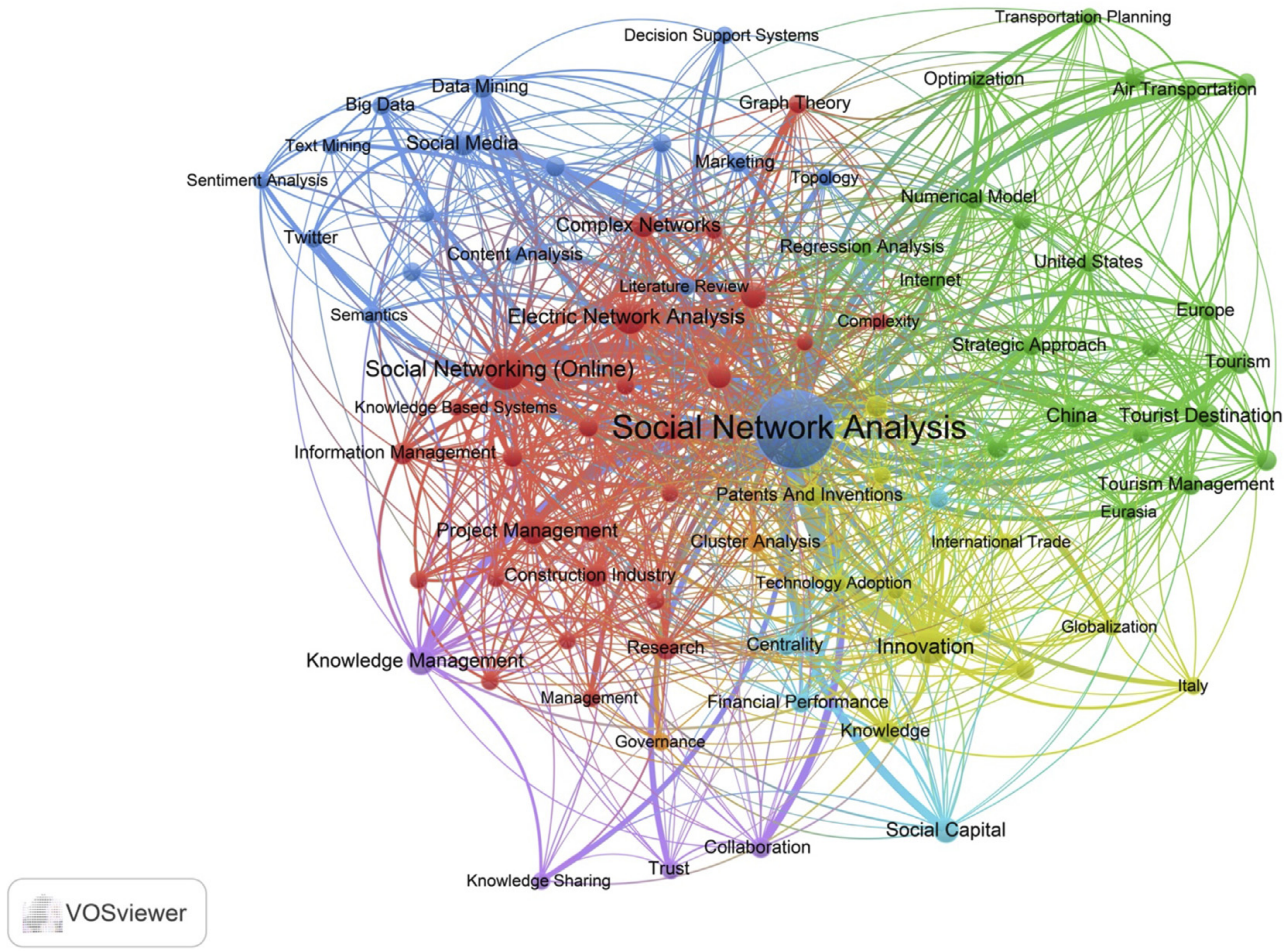
Keywords analysis of SNA in business and management publications.

**Figure 6:**
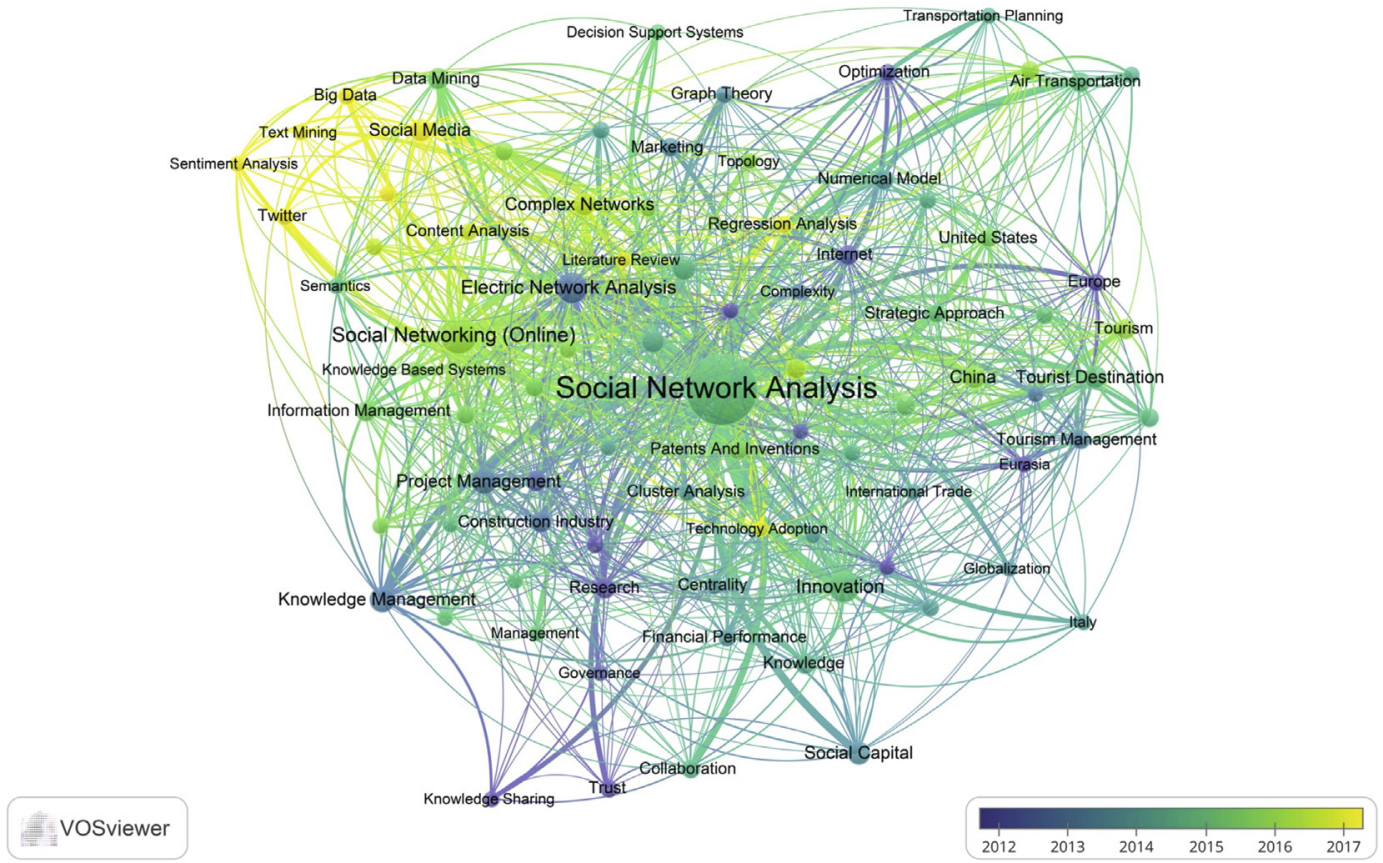
Keywords evolution of SNA in business and management research.

The study conducted a keywords cluster analysis to highlight SNA usage in business and management research and identify how keywords are linked. The keywords cluster analysis was presented in two ways; the first is based on the occurrence level (see Figure 5), and the second is based on the year of publications (see Figure 6). Based on the level of keywords occurrence, SNA research was classified into six clusters: cluster 1 (red nodes) covered research in construction, project management, and information management; cluster 2 (green nodes) covered research in transportation and tourism management; cluster 3 (dark blue nodes) covered research in semantic, big data, and decision support system; cluster 4 (yellow nodes) included research in innovation, international trade, and globalisation; cluster 5 (purple node) explored research in knowledge management and knowledge sharing; cluster 6 (light blue nodes) included research in social capital, and financial performance and management.

According to publication years, the study discovered that from 2012 to 2014, the most frequent keywords were "project management", "optimisation", "technology transfer", and "construction industry". From 2015 to 2016, the keywords shifted to "data mining", "information management", "decision-making", "tourist destination", "air transportation", "airline industry", and "innovation". Recently, "sentiment analysis", "text mining", and "big data" became popular in SNA research.

## 4 Discussion

The study was conducted to analyse SNA research in business and management subjects. Generally, an upward trend was found in the number of publications, significantly increasing since 2005. A significant increase was also discovered in 2020, a 40.3% increase from the previous year, the second-highest increase in the past 20 years. A similar Bibliometric analysis on SNA research without subject limitation had a similar pattern with the study, whereby SNA publications increased gradually since 2005 Based on quality metrics, articles published in 2001 and 2002 had the highest average citations per publication. Moreover, several articles published in those years also had the highest number of citations published in organisational science.

Tsai (2002) articles had the highest citation number (c = 1,124), followed by Reagans and Zuckerman (2001), with 1009 citations. Tsai (2002) displayed intra organisational as a set of social networks and examined networks of collaborative and competitive ties within the organisation. Each unit collaborates for knowledge sharing and competes for resources and market share. Additionally, the centrality concept was used to measure the ability of intra organisational units in the knowledge sharing behaviour. In the same journal, Reagans and Zuckerman (2001) employed the SNA approach to examine the relationship between team density and heterogeneity to its performance using two network metrics: network density to assess communication frequency between team members, and network heterogeneity to explore time allocation of scientists to colleagues far removed in the team tenure distribution. According to above explanation, SNA can be used at different organisation levels, one in unit levels-organisation, and another in person-level interactions under one team.

The study revealed the US, the UK, and China as the most productive countries. Moreover, the study had similar findings as in Su et al. (2019); they found that the US had dominated the research in SNA, UK ranked second, and followed by China. Based on the average c/p, institutions from Asia such as South Korea, China, and Taiwan (average c/p = 23.77) received lower scores than European institutions (average c/p = 28.31). Better quality and impactful research are needed for authors from Asian institutions. The following section describes SNA usage in business and management themes.

### 4.1 Research themes

The SNA is a set of formal methods for studying social structures according to graph theory. Individuals and social actors, such as groups and organisations, are shown in points and their social relations in lines (Korom, 2015). Meanwhile, the structure relations and the location of individual actors have substantial behavioural consequences for individuals and social structure as a whole (Wellman, 1988). The SNA has become a multidisciplinary endeavour extending beyond sociology and social anthropology sciences and to many other disciplines, such as politics, epidemiology, communication science, and others. The next section explores the use of SNA in business and management discipline from publication records between 2001 to 2020 by analysing the keywords co-occurrences.

### 4.2 Project management

The first paper that employed SNA in project management was published in 1997 by Loosemore, believing that construction project participants are embedded in complex social networks that are constantly changing. Furthermore, Loosemore (1997) analysed communication efficiency in the engineering project organisation during crises. Besides, Pryke (2004) had the highest citations in the construction and project management area. Contrary to Loosemore, who applied network analysis at the individual level, Pryke examined the relationship between project actors at the firm level. Pryke also used network analysis in the comparative analysis of procurement and project management of construction projects. Subsequently, SNA publication in project management literature increased substantially.

Chinowsky, Diekmann, & O'Brien (2010) summarised the use of network analysis in project management research. After examining communication efficiency, network analysis analysed networks in relationship-based procurement, the effect of centrality on project coordination, the effect of cultural diversity on project performance, collaboration effectiveness to achieve high-performance teams, and others. The study discovered several popular keywords in project management research, such as "communication", "decision-making", "stakeholder", "accident prevention", "scheduling", "information exchange", "collaborative projects" and others, which explained the use of SNA in this subject. The top three journals publishing SNA usage in project and construction management were the Journal of Construction Engineering and Management (ASCE), International Journal of Project Management (Elsevier), and Construction Management and Economics (Taylor & Francis).

### 4.3 Risk management

The SNA publications in risk assessment and management usually relate to other subjects, such as project management, SCM, knowledge management, and others. The study found 42 related articles, mostly on construction and project management. Notably, SNA improved the effectiveness and accuracy of stakeholder and risk analysis in green building projects (Yang et al., 2016). The model considered the risk associated with stakeholders and the interdependencies of risks for better decision-making. Additionally, Li et al. (2016) employed SNA for risk evaluation and risk response processes in construction projects.

The SNA effectively evaluates the potential risk contributing to schedule delays in project processes by removing key nodes and links, ultimately removing stakeholder risk that is highly interconnected to another risk. Similar to Li, Yu et al. (2017) specifically utilised SNA for social risk in urban redevelopment projects during the housing demolition stage with an identical process. The approach extends beyond construction and project management subject and any other subjects that employed risk assessment or evaluation in their risk management process.

### 4.4 tourism management

The SNA approach in tourism-related subjects was widespread, with "tourist destinations" and “tourism” among the most frequent and top 25 keywords (see Table 2). Besides, Baggio, R. was the most productive author who used SNA in tourism research. Three research streams are related to network analysis in the travel industry setting: the Bibliometric analysis on research collaboration and knowledge creation; network analysis on the travel industry supply, destination, and policy systems; and tourist movements and behavioural patterns (Liu et al., 2017). Besides, the study discovered three significant journals with the most publications in tourism research: Annals of Tourism Research, Tourism Management, and Current Issues in Tourism, similar to Casanueva et al. (2016); whereby tourism management was the most productive journal. Recently, the Annals of Tourism Research surpassed Tourism Management.

Most tourism supply and destination research highlighted collaboration and partnership among tourism stakeholders. Baggio, Scott and Cooper (2010) applied network analysis to explain the topology of stakeholders in Elba, Italy's tourism organisations (hotels, travel agencies, associations, public bodies, and others). The tourism stakeholders were described in quantitative (network metrics) and qualitative (figure) ways. For instance, the percentage of non-connected networks described the sparseness of a network and showed a low degree of collaboration or cooperation between stakeholders. Nonetheless, Leung et al. (2012) and Asero et al. (2016), and others studied tourist movement and behavioural patterns by analysing tourists' itineraries with traditional (interview or online travel diaries) or more-advanced technologies (geographic information system (GIS), global positioning system (GPS), timing systems, camera-based systems, and others). The primary objective is to analyse the main tourist attraction, main tourism movement patterns and change patterns in tourist attractions.

### 4.5 Supply chain management

The use of SNA in supply chain management research had developed in 2010, and numerous scholars were unaware of the possibilities of the SNA approach in the SCM field (Wichmann and Kaufmann, 2016). The supply chain is a network of companies comprising interconnected actors, such as suppliers, manufacturers, logistic providers, and customers (Bellamy et al., 2014). Three journals with the most SNA articles in SCM are the International Journal of Production Economics, International Journal of Production Research, and Journal of Operations Management.

Y. Kim et al. (2011) employed SNA to analyse the structural characteristics of supply networks in a buyers-suppliers network of automotive industries. The networks in the supply chain were classified into the material flow (supply load, demand load, and operational criticality) and the contractual relationship between actors (influential scope, informational independence, and relational mediation). The study discovered that network metrics could be used to analyse the characteristic of supply network structures.

The SNA can also measure and reduce supply chain complexity (Allesina et al., 2010), a concept based on ecological theory. Additionally, eight entropic performance indexes were used: total system throughput, average mutual information, development capacity, overhead in input, export, and dissipation, etc. Contrarily, Ting & Tsang (2014) utilised SNA to identify the possibility of counterfeit products from infiltrating into the supply chain using the transaction records history to detect problematic parties and their suspicious trails. Three SNA measures were included in the study: degree centrality, betweenness centrality, and closeness centrality.

### 4.6 Knowledge management

The SNA usage in knowledge management varies; for instance, Parise (2007) used SNA for knowledge management of human resources, including knowledge creation and innovation, knowledge transfer and retention, and job succession planning. The SNA in knowledge creation and innovation is used to identify the flow of ideas and bottlenecks in the decision-making process. The SNA is also applied to analyse the structure of regional knowledge in the technology specialisation (Cantner et al., 2010), knowledge transfer analysis on sustainable construction projects (Schröpfer et al., 2017), predicting and evaluating future knowledge flows in insurance organisations (Leon et al., 2017) and knowledge transfer from experts to newcomers (Guechtouli et al., 2013), and others. The top productive journals are the Journal of Knowledge Management, Technological Forecasting and Social Change, and the Journal of Construction Engineering and Management.

### 4.7 Technology and innovation management

The SNA is used for innovation and technology transfer. Keywords under this subject include "citation "innovation", "patents and inventions", "technological development", and others. The SNA metrics utilised to assess the performance and centrality of individuals in virtual research and design (R&D) groups by analysing their e-mails (Ahuja et al., 2003), identifying the position and relationships between innovators (Cantner and Graf, 2006), research collaboration network between university-industry (Balconi and Laboranti, 2006), and others.

The SNA in patents and inventions identify companies with a significant legal influence on the applied technologies by analysing intellectual property lawsuits between companies (H. Kim and Song, 2013). Patents data were also popular to study technological innovation of electronic companies; SNA was employed to cluster the patents and find vacant technology domains (Jun and Sung Park, 2013). Furthermore, patent data in SNA enables exploring the technology evolution of certain products (Lee et al., 2010). Specifically, the top three journals were: Technological Forecasting and Social Change, Technological Analysis and Strategic Management, and Industry and Innovation.

## 5 Conclusion

After SNA was introduced in 1969 by Mitchell, many researchers from various fields were interested in studying the relationship between nodes. The most frequent disciplines that used SNA are sociology, anthropology, social psychology, and communication. Nevertheless, SNA usage in business and management discipline was limited. Hence, the study analysed the trend and performance of SNA in the business and management discipline from 2001 to 2020. The study revealed a steady upward trend of publications in this field and increased significantly since 2005. The US, the UK, and China were the most productive countries. Although the study found three Asian institutions as the most productive countries, the average c/p was lower than the European and American countries. Besides, SNA as a research tool has been published in multidisciplinary journals, ranging from Journal of Management in Engineering to Journal of Knowledge Management, depending on the subject of investigation.

The study also performed a co-occurrence keywords analysis to examine the research cluster and emerging research topics in SNA, especially in business and management studies. The study revealed six clusters, each containing one to two research disciplines. The SNA has been employed in numerous topics, including project management, risk management, tourism management, supply chain, knowledge management, and technological management. Observably, big data, social media and sentiment analysis are the trending topic in SNA.

The research contributions include: first, the publication trend and research productivity show the current issue and development of SNA in the business and management discipline; second, the data on most productive authors and institutions academic communication and cooperation among scholars in related fields; and lastly, the visualisation of research topics mapping and the cluster analysis explored the current use of SNA in different discipline and formulated future research agenda.

The study limitations are: first, the study only considered the Scopus database and SNA literature could be more extensive. Other significant databases, such as WoS and CNKI (Chinese National Knowledge Infrastructure) should be considered for future research. Second, in the co-occurrence keywords analysis, a threshold was set to limit important keywords; thus, the study might not include several research topics using SNA. Third, although the study has cleaned the database, titles not purely from business and management disciplines might be included due to journal sources with multi subject classification. Lastly, we only employed standard bibliometric measures as a quantitative assessment in this research; the inclusion of SNA’ centrality index can be considered in future study to assess the power and importance of authors, institutions, countries, and journals.

## Institutional review board statement

Not applicable.

## Informed consent statement

Not applicable.

## Decalarations

### Author contribution statement

All authors listed have significantly contributed to the development and the writing of this article.

### Funding statement

This research did not receive any specific grant from funding agencies in the public, commercial, or not-for-profit sectors.

### Data availability statement

Data included in article/supplementary material/referenced in article.

### Declaration of interests statement

The authors declare no conflict of interest.

### Additional information

No additional information is available for this paper.
